# Caregivers’ experiences and challenges of the diagnostic odyssey in Dravet syndrome

**DOI:** 10.1186/s13023-025-03772-7

**Published:** 2025-05-16

**Authors:** Jan Domaradzki, Dariusz Walkowiak, the Association for People with Severe Refractory Epilepsy Dravet.pl

**Affiliations:** 1 Department of Social Sciences and Humanities, Poznan University of Medical Sciences, Rokietnicka 7, Poznań, 60-806 Poland; 2 Department of Organization and Management in Health Care, Poznan University of Medical Sciences, Poznan, Poland; 3 ul. Starowapiennikowa 8/1, Kielce, 25-112 Poland

**Keywords:** Diagnostic delay, Diagnostic odyssey, Dravet syndrome, Family caregivers, Parent survey, Parents experiences, Rare genetic epilepsy

## Abstract

**Background:**

Although the genetic background of Dravet syndrome (DS) has been determined and is clearly described, and genetics tests that support a clinical diagnosis are available, DS diagnosis is often based on the clinical assessment alone, which may lead to a late or missed diagnosis. This study explores experiences of caregivers’ of persons with DS with the diagnostic odyssey and their perception of its consequences for DS patients.

**Results:**

106 family caregivers connected with the Association for People with Severe Refractory Epilepsy DRAVET.PL completed an anonymised, self-administered, computer-assisted online survey on parents’ experiences of the diagnostic journey conducted from March to June 2024. Although 96.2% of DS parents reported that their children experienced initial symptoms in the first year of life, 58.4% indicated that it took more than a year before DS was diagnosed and 72.7% reported that their DS child received at least one misdiagnosis. While 6.6% of patients were diagnosed by the first doctor consulted, 65.1% had to consult between two and four specialists and 22.8% consulted more than five specialists. 19.8% of parents confirmed that they sought diagnosis abroad. 58.4% of DS parents suggested that delayed diagnosis was harmful to their children’s health. Many believed that it resulted in taking unnecessary or inappropriate medications (67%), hospitalisations (32.1%), or medical interventions (15.1%). Many parents reported problems with accessing genetic counselling and psychological support.

**Conclusions:**

Since DS parents report the multifaceted and protracted diagnostic journey in their children, underscoring the duration of the diagnostic process, numerous misdiagnoses and the number of healthcare professionals involved in achieving the confirmed DS diagnosis, this study highlights the need for widespread access to genetic testing, which usually concludes the diagnostic odyssey and is crucial for managing the proper treatment plan for DS patients. It also shows the need to increase general practitioners’ awareness of the developmental and epileptic encephalopathy (DEE) and the creation of more rapid and transparent referral procedures for children with DEE.

## Background

Dravet syndrome (DS; ICD-10: G40.834, ICD-11: 8A61.11, ORPHA: 33069, OMIM: 607208) is a severe, genetically based developmental epileptic encephalopathy that usually begins in infancy or early childhood after a normal prior development [1]. While first described in France in 1978 by Charlotte Dravet as severe myoclonic epilepsy of infancy (SMEI), it was added to the International League Against Epilepsy (ILAE) classification as a separate syndrome in 1989 [2]. DS is characterised by prolonged and recurrent seizures that are often triggered by high temperatures, fever, physical exercise, emotional stress or excitement [1, 3, 4] and vaccinations [5–7].

While more than 90% of diagnosed DS cases are caused by mutations in the SCN1A gene (locus2q24) encoding the alfa-subunit of voltage-gated sodium channel (Nav1.1), which is required for the proper function of brain cells and controls electrical messages in the brain, most of these mutations arise de novo [8] and are of paternal origin [9, 10]. At the same time, several other genes have also been reported to cause DS-like phenotypes, including SCN2A, SCN8A, SCN9A, SCN1B, PCDH19, GABRA1, GABRG2, STXBP1, HCN1, CHD2, and KCNA2 [11] responding to other than DS treatment [12].

DS is a rare disease with an estimated incidence in the general population between 1:16.000 and 1:40.000 [13], and, according to the International League Against Epilepsy (ILAE), its incidence among children is 6.5:100,000 [14]. The first symptom of DS usually manifests between the ages of 1 and 18 months in a previously healthy child in the form of a convulsive seizure. Still, in the majority of cases, seizures appear between 4 and 8 months. While most frequently, these first symptoms include febrile seizures and convulsive status epilepticus, myoclonic, focal, tonic-clonic, febrile or afebrile, and prolonged generalised hemiclonic seizures are also present. At the same time, while the frequency of seizures increases over time, their duration tends to become shorter [15–17]. All these symptoms lead to refractory epilepsy, neuro-developmental delay, sensory integration disorders [18], speech impairment, aggressive behaviours and social problems. DS, however, is also associated with multiple comorbidities, which include ataxia, autism spectrum disorder, attention and hyperactivity issues, circadian rhythm disorder, eating problems and disturbed sleep, which often worsen during adolescence and persist throughout adulthood [15, 19–22].

Despite recent advances in available treatments [4, 23–27], DS is also distinguished from other forms of epilepsies by its high drug resistance, which impairs patients’ health and caregivers’ feelings of burden. Simultaneously, persons with DS are at significant risk of status epilepticus and sudden unexpected death in epilepsy (SUDEP) [28, 29], which are the two most common causes of premature death among persons with DS [30, 31].

Over the years, due to the progress in molecular diagnostics [4, 23, 32] and increased awareness of DS among paediatric neurologists who are more familiar with its diagnosis and treatment [20], many persons with DS have been diagnosed at younger ages [23–26]. At the same time, despite advances in knowledge on the genetic basis of DS and the availability of genetic testing that helps to confirm the disease, DS often remains a clinical diagnosis based on clinical assessment alone, which may, in turn, lead to a late or missed diagnosis [20, 33–36]. This is of crucial importance because accurate early diagnosis allows for for the establishment of therapeutic plans to help control seizures, reduce the risk of SUDEP and avoid the use of sodium channel blockers that may exacerbate seizures and worsen prognosis [4, 23, 32]. Late diagnosis or misdiagnosis of DS may, moreover, lead to many unnecessary, costly hospitalisations, invasive testing and interventions, and ineffective therapies, including frequent medication changes, which may worsen seizures or status epilepticus and affect a child’s development [20, 33–36]. Additionally, although most genetic variants in DS are *de novo*, in 5–10% SCN1A mutations are inherited as autosomal dominant in families with genetic epilepsy with febrile seizures plus (GEFS+) syndrome with a 50% risk of inheritance, and early diagnosis is essential in genetic counselling to families [16, 37].

While earlier studies in Poland have focused either on the genotype and phenotype heterogeneity of the DS population in the country [8], treatment schemes for DS patients [27] or factors affecting their quality of life [15, 38], much less attention, has been paid to the diagnostic journey in DS. This study therefore aims to explore caregivers’ parents’ perception of the diagnostic odyssey in their DS children, including (1) caregivers’ experiences with the diagnostic process in DS, (2) their perception of the delayed diagnosis, and (3) its consequences for DS patients.

## Materials and methods

### Study design

While this survey was part of a larger study on the experiences of Polish family caregivers of persons with a rare disease (RD), it delves into the experiences of parents of persons with Dravet syndrome related to the diagnostic process. Since there is to date no registry of DS patients in Poland and the exact number of persons affected is unknown, the study was conducted with the support of the patient advocacy organisation the Association for People with Severe Refractory Epilepsy DRAVET.PL (https://www.dravet.pl). It was designed as an anonymised, self-administered, computer-assisted online survey about parents’ experiences with the diagnostic process [39].

## Research tool

Since there is no specific tool assessing caregivers’ experiences with the diagnostic odyssey, an original ad hoc questionnaire was designed for this survey. While it was designed according to the guidelines of the European Statistical System [40], the themes derived from the literature review on the topic. At first, a drafted questionnaire was evaluated by four experts: a paediatrician specialising in RDs, a medical sociologist, a public health specialist and the parent of a DS child. It was then piloted on an online group of five parents and re-evaluated by the same specialists. This led to the reformulation of four items. The association then approved the final version of the questionnaire for people with severe refractory epilepsy DRAVET.PL.

The questionnaire consisted of 28 single-choice, closed-ended questions divided into five sections. The first included queries concerning the socio-demographic characteristics of DS caregivers. The second section asked questions regarding DS patient’s characteristics. The third section included questions regarding the diagnostic process. The fourth section related to caregivers’ experiences with the diagnostic journey in DS. The last section asked questions about DS caregivers’ perception of the implications of delayed diagnosis.

Descriptive definitions were employed instead of technical jargon to make the survey more straightforward to complete. Most questions also allow for the neutral response ‘I do not know”. At the same time, to avoid misunderstandings and to help participants understand the issues discussed, they were provided with several definitions before completing the survey. *Genetic counselling* was therefore described as “a communication process which aims to help to understand how the genetic condition can affect individuals, couples and families affected by a genetic disorder or its risk, and helps to adapt to the medical, psychological, familial and reproductive implications of the genetic conditions”. *Unnecessary or inappropriate medications* were defined as “medications that, in caregivers’ opinions, might have been avoided or prevented by a timely and appropriate diagnosis or were withdrawn after DS was confirmed”. *Unnecessary tests, treatments or surgeries* were defined as “any procedure that, in caregivers’ opinion, might have been avoided or prevented by timely and appropriate diagnosis”. Finally *unnecessary hospitalisations* were defined as “hospitalisations that, in caregivers’ opinion, might have been avoided or prevented by timely and proper diagnoses”.

## Participants and setting

Eligible participants were recruited with the support of the patient advocacy organisation the Association for People with Severe Refractory Epilepsy DRAVET.PL via their page on Facebook. The criteria for participation included being a parent or family caregiver of a person with a confirmed DS, being a provider of direct care for a person with DS and being able to participate in an online survey using electronic devices.

## Data collection

The survey was conducted between March and June 2024 among family caregivers of persons with DS. Firstly, the research co-ordinator contacted the Association for People with Severe Refractory Epilepsy DRAVET.PL. to ascertain whether it was interested in participating in the survey. Once we received their permission, an letter of invitation and the online version of the questionnaire were posted on the Facebook page and all affiliated DS caregivers who met the inclusion criteria were invited to participate in the survey. Once the caregivers provided online written informed consent to participate, the survey was completed via electronic devices, which took between approximately 15 and 17 min. Two follow-up messages were sent in May and June.

## Ethical issues

This survey followed the guidelines of the Declaration of Helsinki (revised in 2000) [41]. It was performed after the ethics and research governance approval were obtained from the Poznan University of Medical Sciences Bioethics Committee (KB – 228/24). Additionally the permission to post a questionnaire on the Facebook page of the Association for People with Severe Refractory Epilepsy DRAVET.PL was also obtained. Before beginning the survey, all study participants were shown the online consent form and asked to check the “I agree” or “I do not agree” box. All caregivers gave their informed written consent.

### Analysis

Descriptive statistics were used to summarise participant responses briefly, with counts and percentages illustrating the distribution of answers. Statistical analysis was performed using JASP 0.18.3. The table results are rounded to one decimal place according to standard mathematical rules, so they may not always add up exactly to 100%.

## Results

Of all 106 caregivers who volunteered and participated in the survey the majority were women (82.1%) and a small minority of men (17.9%), all of Polish origin (Table 1). The relationship with the DS patients was predominantly maternal (81.1%), with fathers representing 17.9% of respondents and grandparents 0.9%. Most caregivers were within the 30–49 age range, 30–39 years (36.8%) and 40–49 years (46.2%).

**Table 1:** Socio-demographic characteristics of DS caregivers

Characteristics	*N* (%)
*Sex*	
woman	87 (82.1)
man	19 (17.9)
*Relationship with a DS patient*	
mother	86(81.1)
father	19(17.9)
grandmother/grandfather	1(0.9)
*Age*	
20–29	4 (3.8)
30–39	39 (36.8)
40–49	49 (46.2)
50–59	14 (13.2)

The distribution between the sexes among DS patients was almost equal, with females constituting 50.9% and males 49.1% (Table 2). The age of DS patients ranged from 0.9 to 35 years, with a mean age of 10.8 years (SD = 6.5) and a median age of 10 years. The onset of the first symptoms predominantly occurred between 6 and 12 months of age (52.8%) and between 2 and 5 months of age (41.5%). Only a small number of patients showed symptoms in the neonatal period (1.9%) or between 1 and 3 years of age (3.8%).

The initial symptoms that prompted the diagnostic process were primarily neurological (97.2%). Only three caregivers (2.8%) described these symptoms as non-neurological; in two of these cases, however, they could likely be interpreted as neurological. In addition to neurological symptoms, some parents also reported other accompanying symptom groups. These included immunological (2.8%), cardiovascular (1.9%), nephrological (1.9%), gastrointestinal (0.9%), motor (3.8%), cognitive (1.9%), general developmental (6.6%) and other symptoms (2.8%).

**Table 2:** DS patients’ characteristics

Characteristics	*N* (%)
*Sex*	
female	54 (50.9)
male	52 (49.1)
*Age (in years)*	
Range	0.9–35
Mean(SD)	10.8 (6.5)
Median	10.0
*A time when the first symptoms appeared*	
The neonatal period (first month of life)	2 (1.9)
2–5 months of age	44 (41.5)
6–12 months of age	56 (52.8)
1–3 years of age	4 (3.8)
Type of symptoms that initiated the diagnostic process*	
neurological	103 (97.2)
immunological	3 (2.8)
cardiovascular	2 (1.9)
nephrological	2 (1.9)
gastrointestinal (digestive)	1 (0.9)
motor	4 (3.8)
cognitive	2 (1.9)
general development	7 (6.6)
other	3 (2.8)

* It was possible to give more than one answer

As for parents’ experience of the diagnostic journey, the study showed three important findings (Table 3). Firstly, most caregivers reported the DS diagnosis in their child was confirmed between the first and the third year of life (45.3%). Additionally, 22.6% stated that the person they were caring for was diagnosed with DS between 6 and 12 months of life. 22.7% of parents, however, declared that a DS diagnosis was made between ages four and ten and 8.5% after age 11.

Secondly, while 20.8% of caregivers reported receiving the DS diagnosis up to 6 months after the onset of the initial symptoms, and for 20.8% it was between 7 and 12 months, a substantial portion reported experiencing a prolonged diagnostic journey, which for 22.6% of patients lasted between 1 and 2 years, for 19.8% between 3 and 5 years and 14.4% more than 6 years. While 27.4% of parents reported no misdiagnoses before DS was confirmed, 43.4% of patients faced one misdiagnosis and 27.4% received 2 to 3 misdiagnoses. Four of all 77 caregivers who reported receiving one misdiagnosis or more neglected to specify the misdiagnosis. The remaining 73 caregivers provided examples of 20 different conditions incorrectly diagnosed in those they were caring for. In some cases, caregivers indicated multiple misdiagnoses but provided only one specific example. Altogether, these 73 caregivers identified 108 instances of misdiagnosis or incomplete diagnoses. The most frequently mentioned conditions were febrile seizures (*n* = 34), epilepsy (*n* = 30) and drug-resistant epilepsy (*n* = 13).

Finally, many parents faced the challenge to consult several doctors before the DS diagnosis was confirmed. While only 6.6% of respondents reported that the first doctor consulted diagnosed DS, 65.1% reported visiting between 2 and 4 doctors, 22.6% between 5 and 7, and 5.7% more than 8.

**Table 3:** Diagnostic odyssey in DS

Characteristics	*N* (%)
*Age when the DS diagnosis was made*	
2–5 months of age	1 (0.9)
6–12 months of age	24 (22.6)
1–3 years of age	48 (45.3)
4–5 years of age	13 (12.3)
6–10 years of age	11 (10.4)
11–15 years of age	6 (5.7)
16–18 years of age	2 (1.9)
over 18	1 (0.9)
*How long did it take before the DS diagnosis was confirmed?*	
1–3 months	4 (3.8)
4–6 months	18 (17)
7–12 months	22 (20.8)
1–2 years	24 (22.6)
3–5 years	21 (19.8)
6–7 years	4 (3.8)
8–10 years	7 (6.6)
11–15 years	3 (2.8)
16–20 years	2 (1.9)
over 20 years	1 (0.9)
*The number of misdiagnoses received before the DS diagnosis was confirmed*	
0	29 (27.4)
1	46 (43.4)
2–3	29 (27.4)
4–5	2 (1.9)
*Type of misdiagnosis or incomplete diagnoses a person received before DS diagnosis was made*	
Febrile seizures	34
Epilepsy	30
Drug-resistant epilepsy	13
Idiopathic generalised epilepsy	4
Infantile epileptic spasms syndrome (IESS)	3
Infantile epilepsy	3
Lennox-Gastaut syndrome	2
Cerebral palsy	2
Viral encephalitis	2
Doose Syndrome	1
Muscle cramps	1
Autism spectrum disorder	1
hypotonia	1
Homocystinuria	1
Hypoxemia	1
Aphasia	1
Primary immunodeficiency	1
Unspecified metabolic disorder	1
Juvenile idiopathic arthritis (JIA)	1
Caccine adverse event (VAE)	1
missing answer	4
*The number of physicians consulted before the DS diagnosis was confirmed*	
1	7 (6.6)
2–4	69 (65.1)
5–7	24 (22.6)
8–10	4 (3.8)
11–15	2 (1.9)

Table 4 presents DS caregivers’ experiences of the diagnostic journey. It shows that, while most DS patients were referred for diagnostic tests by a neurologist (73.6%), 15.1% of parents sought diagnostics alone, while only 2.8% were referred by their family doctor. Significantly, 19.8% of caregivers sought diagnostic assistance abroad.

As for diagnostics itself, genetic panels were the most commonly reported (78.3%), followed by Whole Exome Sequencing (WES) (9.4%) and other tests (8.5%). While 67.9% of caregivers reported that their DS relatives had not undergone WES testing, among those who had, the majority indicated WES trio (17%), followed by WES single (10.4%) and WES quarto (4.7%). Simultaneously, 26.4% of respondents reported that a WES genetic test confirmed a previously made DS diagnosis in their relative. The primary sources of information about WES testing indicated by the caregivers were support groups for parents (35.8%), the Internet (17.9%) and other parents/caregivers (14.2%). Meanwhile, only 18.8% knew about it from healthcare professionals, including a specialist from a hospital or a specialist as the result of a private visit (8.5% apiece). In terms of financing, nearly half the caregivers reported that the National Health Fund reimbursed them for the diagnostic tests for their DS relative (47.2%), while 32.1% had to finance them through private means. Additionally, 14.2% declared that diagnostic tests were covered through participation in experimental research or hospital grants.

84.9% of caregivers declared that at some point during the genetic testing procedure, they had received psychological advice or counselling. On the other hand, of 67% of caregivers who reported having had contact with a genetic clinic, 59.4% declared receiving genetic test results. In comparison, 28.3% received a diagnosis, and even fewer received genetic counselling (21.7%), and only 0.9% of parents reported receiving psychological support.

**Table 4:** DS caregivers’ experiences with the diagnostic journey

	*N* (%)
*The doctor who referred a child for a diagnostic test*	
a doctor from the hospital where the baby was born	4 (3.8)
a family doctor	3 (2.8)
paediatrician	5 (4.7)
another specialist (e.g. neurologist, gastroenterologist, geneticist)	78 (73.6)
no one; we began the diagnostics ourselves	16 (15.1)
*Have you sought help abroad at any stage of diagnosis?*	
yes	21 (19.8)
no	85 (80.2)
*Test that led to the diagnosis*	
EEG	2 (1.9)
aCGH microarrays	1 (0.9)
genetic panel	83 (78.3)
Whole Exome Sequencing (WES)	10 (9.4)
SCN1A testing	1 (0.9)
other unspecified genetic testing	9 (8.5)
*Did your child have a Whole Exome Sequencing test performed?*	
WES single	11 (10.4)
WES duo	0 (0)
WES trio	18 (17)
WES quarto	5 (4.7)
no	72 (67.9)
*Did the WES genetic test confirm the diagnosis that was previously made?*	
WES test was not performed	69 (65.1)
yes	28 (26.4)
no	9 (8.5)
*How did you find out about the WES test?*	
a family doctor at a clinic during a visit under the National Health Fund	1 (0.9)
a doctor during a the National Health Fund visit	1 (0.9)
a specialist in a hospital during the National Health Fund tests	9 (8.5)
a specialist during a private visit	9 (8.5)
The Internet	19 (17.9)
other parents/caregivers of persons with a rare disease	15 (14.2)
a support group for parents	38 (35.8)
I’ve never heard of this type of test	14 (13.2)
*Source of financing for diagnostic tests*	
reimbursement from the National Health Fund	50 (47.2)
private financial resources	34 (32.1)
help from family, relatives, friends	1 (0.9)
support from foundations and associations	3 (2.8)
‍ participation in experimental research	2(1.9)
public collection	1 (0.9)
participation in experimental research, university/hospital research grant	15 (14.2)
*Did you receive psychological advice or counselling at any stage of diagnosis?*	
yes	90 (84.9)
no	16 (15.1)
*Did you have contact with a genetic clinic during the diagnosis?*	
yes	71 (67)
no	35 (33)
What did you get from contacting the genetic counselling centre? *	
we had no contact with the genetic counselling centre	14 (13.2%)
genetic test results	63 (59.4)
diagnosis	30 (28.3)
genetic counselling	23 (21.7)
psychological support	1 (0.9)
other	13 (12.3)

* It was possible to give more than one answer

1. 58.4% of caregivers believed that delayed diagnosis was detrimental to their DS relatives’ health (Table 5). 67% declared that misdiagnosis or late diagnosis resulted in taking unnecessary or inappropriate medications. 50.9% of parents mentioned between 1 and 3 medications and 11.3% between 4 and 6, and 6.6% more than 7.
2. 32.1% of respondents also suggested that misdiagnosis or late diagnosis resulted in unnecessary hospitalisations. Of those, 16% mentioned between 4 and 6 unnecessary hospital stays, 15.1% between 1 and 3, and 7.5% more than seven.

Unnecessary tests, treatments and surgeries were also mentioned by 15.1% of parents, 11.3% of caregivers suggesting between 1 and 3 such procedures, and 8.5% suggesting more than 4.

**Table 5:** DS caregivers’ perception of delayed diagnosis

	*N* (%)
*In your opinion, has the delay in correct diagnosis been harmful to your relative’s health?*	
definitely yes	38 (35.8)
rather yes	24 (22.6)
rather no	24 (22.6)
definitely no	5 (4.7)
I do not know	15 (14.2)
*In your opinion, has your relative taken unnecessary/inappropriate medications as a result of previous inappropriate diagnoses?*	
yes	71 (67)
no	25 (23.6)
I do not know	10 (9.4)
*In your opinion, how many unnecessary medications has your relative taken as a result of previous incorrect diagnoses?*	
not applicable	33 (31.1)
1–3	54 (50.9)
4–6	12 (11.3)
7–10	5 (4.7)
11–20	2 (1.9)
*In your opinion, was your DS relative unnecessarily hospitalised as a result of previous inappropriate diagnoses?*	
yes	34 (32.1)
no	55 (51.9)
I do not know	17 (16)
*In your opinion, how many times has your DS relative been unnecessarily hospitalised as a result of previous inappropriate diagnoses?*	
not applicable	65 (61.3)
1–3	16 (15.1)
4–6	17 (16)
7–10	1 (0.9)
over 10	7 (6.6)
*In your opinion, has your DS relative undergone unnecessary tests, treatments or surgeries as a result of previous, inappropriate diagnoses?*	
yes	16 (15.1)
no	70 (66)
I do not know	20 (18.9)
*In your opinion, how many unnecessary tests, treatments or surgeries has your DS relative undergone as a result of previous inappropriate diagnoses?*	
not applicable	85 (80.2)
1–3	12 (11.3)
4–6	3 (2.8)
7–10	2 (1.9)
over 10	4 (3.8)

## Discussion

The genetic background of DS has been determined and well reported [8–11], and genetics tests that reveal a mutation to support a clinical diagnosis are available [4, 23, 32]. Many doctors, however, including family doctors, paediatricians and even neurologists in Poland, appear unfamiliar with this syndrome.

This is the first study to quantify the experiences of Polish DS caregivers with the diagnostic odyssey and its perceived consequences and it reports three significant findings. Firstly, it shows that, while most persons with DS experienced initial symptoms early in life (94.3%) and 23.5% reported that the DS diagnosis was made before the age of one year, many faced a diagnostic odyssey and a labyrinth. While 23.5% of parents reported receiving a diagnosis within 1 year of their child’s life, 45.3% were diagnosed between 1 and 3 years, 22.7% were diagnosed between the ages of 4 and 10, and 8.5% after the age of 11. Although 41.6% of DS patients were diagnosed within a year of the onset of the initial symptoms and 22.6% within 1 and 2 years, according to 19% of caregivers, it took between 3 and 5 years and in the case of 14.1% more than 6 years. A previous Polish study conducted by Paprocka et al., however, documented that, although 18.9% of DS children were diagnosed within the first 12 months of life, 83.63% were diagnosed within the first 6 years [15]. Another Polish survey demonstrated that although 18.6% of parents reported that the diagnostic process in their DS children lasted less than a year, for 54.6%, it took up to 3 years and for 5.4%, it was more than 10 years [42].

This aligns with other studies that show that DS families face many challenges during the diagnostic journey. Bremer et al., for example, demonstrated that, although in DS children born after 2003 the average time from seizure onset to diagnosis was 2.1 years, in all Norwegian DS children this period was 7.4 years (1–15 years) [33]. A multicentre study conducted in the United States also showed that DS patients often experience delayed diagnosis, which on average lasted 4.8 years from the first seizure until the final diagnosis [34]. Similarly, Skluzacek et al. reported that for more than 50% of patients the diagnosis was delayed for over 3 years, while for 23%, it was over 5 years and for 8% over 10 years [43]. In Belgium, Lagae et al. reported that, although in 88% of infants DS diagnosis was made within the same year the initial seizure occurred (until 12 months of age), in 83% of adults and less than 20% of older children aged between 6 and 11, undiagnosed in the first instance, the DS diagnosis took 4 or more years [20]. Similarly, Nabbout et al. documented that in a group of 20 children (12 girls and 8 boys) the mean age at diagnosis was 2 years and 10 months. At the same time, it was much shorter than in other countries: in the UK, diagnosis took 4 years and 2 months and in the USA 5 years; in Italy, it was 1 year and 1 month and in Australia, 2 years [35]. Finally, in a group of adults with complex epilepsy of unknown cause and otherwise undiagnosed who underwent WGS testing the median age was 44.5 years (range 28–52), and the median age of onset of developmental delay was 2.5 years (range 1.25-4) [36].

This should come as no surprise, since the diagnostic odyssey in epilepsy and RD patients alike is well documented [44–48]. Libura et al., for example, reported that 53.1% of patients with RD in Poland received the final diagnosis a year after the initial symptoms presented. In 8.9% of cases it took 10 years or more [49]. Similarly, Polish caregivers of persons with Huntington’s disease have reported that the mean time for diagnosis is 10.5 years (range 1–30) [50]. In Australia 38% of parents of RD children consulted between 3 and 5 doctors before the final diagnosis was made, and 14% visited between 6 and 10 doctors. Additionally, 43% felt the diagnosis was delayed [51]. A study by Grier et al. found that on average patients with mitochondrial diseases saw 8.19 clinicians and 54.6% received at least one nonmitochondrial misdiagnosis before their final mitochondrial diagnosis [52]. Similarly, Benito-Lozano et al. showed that the mean time for receiving an RD diagnosis in Spain was 6.18 years, and more than half of RD patients experienced diagnostic delay: in 56.4% of patients it took over a year, in 19% between 1 and 3 years, 16.7% between 4 and 9 years, and 20.9% waited for more than 10 years to be diagnosed. At the same time, it was shown that patients with neurological diseases were more likely to be subject to diagnostic delay [53]. In Germany RD patients waited on average 4.4 years between the initial symptoms and the final diagnosis. During that time, they would have visited 6 doctors or hospitals before consulting an expert, which would take approximately 1,546 days before a patient was referred to the expert [54]. Finally, a recent European survey conducted among 6,507 people living with 1,675 RDs in 41 countries showed that the average time for diagnosis was 4.7 years [55].

Secondly, this research also found that, while DS parents often face an uncertain prognosis, they also struggle with several misdiagnoses and visit numerous doctors before DS is confirmed. As more than 70% of parents reported that their children received at least one misdiagnosis, this research highlights the diagnostic challenges and complexities related to DS. This is confirmed by the fact that less than 7% of DS patients were diagnosed by the first doctor consulted, over 65% of parents reported visiting between 2 and 4 specialists, and 22.8% consulted more than five specialists. Previous studies, however, also showed that only 2.7% of Polish DS parents reported receiving a final diagnosis in their children after consulting only one doctor, while 37.3% visited between 4 and 6, and 16.1% consulted six or more [42]. Similarly, a multinational survey conducted by Skluzacek et al. demonstrated that 68% of DS families consulted 3 or more neurologists before the final diagnosis was made, and 29% consulted five or more. In 46% of cases, it was, moreover, not a doctor who first suggested a DS diagnosis [43].

Again, this is in line with previous findings describing the diagnostic process in RDs as an odyssey and a complex labyrinth filled with wrong turns and dead ends in which it is difficult to find one’s way [56, 57]. A previous Polish study reported that while 61.4% of patients with RDs received at least one misdiagnosis, on average they received 3.5 [49]. Similarly, a recent UK study documented that 71.6% of RD patients were misdiagnosed before receiving the final diagnosis. Before receiving the final diagnosis, 61.7% reported visiting on average, 4 or more specialists and 13.6% consulted more than 10 [58]. In Australia 30% of adults with RD reported that their diagnosis was delayed by five years or more, 66% consulted three or more specialists before the final diagnosis and 45.9% received at least one misdiagnosis [59]. Similarly, Zurynski et al. demonstrated that 38% of Australian parents of children with RD reported consulting six or more doctors before receiving the final diagnosis, 37% suggested that the diagnosis was delayed and 27% reported receiving a misdiagnosis [60]. A European survey documented that 22% of RD patients consulted at least 8 healthcare professionals and 73% of patients were misdiagnosed at least once [55].

What is equally important is that almost 20% of DS parents enrolled in this study sought diagnosis either abroad (19.8%) or by themselves (15.1%), therefore supporting previous findings that parents who experience diagnostic delays in their RD children often use internet search engines as an efficient diagnostic tool [61].

Thirdly, this study shows that almost 60% of DS parents believed that prolonged diagnosis had a deleterious effect on their children’s health and many others complained that the diagnostic odyssey resulted in numerous unnecessary or inappropriate hospitalisations, medical procedures and medications. Many parents also reported problems with accessing genetic counselling and psychological support. Bremer et al., however, also reported that 68% of DS children underwent unnecessary non-invasive and invasive examinations and were evaluated for epilepsy surgery (video-EEG monitoring over several days, MRI investigation under general anaesthetic or invasive examination with intracranial EEG-electrodes) before the final diagnosis was made [33]. Bluvstein & Wenniger reported that delayed diagnosis in a DS boy, which took almost 7 years, resulted in numerous unnecessary hospitalisations, invasive and harmful interventions, including brain surgery and a strip band study, and taking a “cocktail” of several seizure medications, which in turn lead to harmful side effects and overdoses [62]. Similarly, Swedish caregivers who complained at the delays in the diagnostic process were also frustrated that it forced them to take responsibility for the choice of medication [63].

Similarly, Australian parents of children with RD expressed concerns related to the unknown effects of treatments and healthcare management approaches [64]. Another study of 30 Australian children with RD documented that delayed diagnosis resulted in 168 visits to general practitioners and 260 visits to specialist doctors [51]. A Spanish study demonstrated that persons with a diagnostic delay had to travel more often and greater distances, including to other provinces or another countries. They also reported more specialist visits, tests performed, hospitalisations and surgical interventions, and had to change their treatment more often [65]. Finally, according to a European survey, 72% of RD patients reported that delayed diagnosis resulted in deterioration of their health, 52% received inappropriate care, treatment or surgery, and 68% had delayed access to appropriate care, treatment or surgery [55]. Similarly, Willmen et al. reported that RD patients in Germany took on average 25 examination appointments outside the expert centre. Although some had as many as 109 examination days, no diagnosis was made in 79% of cases [54].

At the same time, previous research demonstrated that one of the most important factors affecting diagnostic delay in rare diseases is a lack of or inadequate knowledge among healthcare professionals, including general practitioners, who receive little training on such diseases during their undergraduate or postgraduate years [66–70]. This is confirmed by the number of caregivers enrolled in this study who reported receiving several misdiagnoses of their DS relative and having to visit numerous specialists before the DS diagnosis was made. It is equally important to stress that, while some DS patients were misdiagnosed, many parents report that their children receive incomplete diagnosis (either epilepsy or drug-resistant epilepsy), which may have led to neglect of examination of possible genetic causes. This in turn may result in patients health needs not being met, either by limiting treatment options specific to DS or by increasing the risk of the intake of harmful drugs and hindering the monitoring of their complications. Silvennoinen et al. correctly point out that all people with epilepsy of undiagnosed cause or refractory epilepsy should have genetic testing [36].

On the other hand, it was shown that delayed diagnoses is more probable when the symptoms appear in childhood or adolescence [53, 55]. This survey is also in line with previous studies showing that diagnostic delay is particularly common among neurological patients [52–55]. Finally, the diagnostic odyssey is affected by the need to travel to a different specialist or hospital far from their homes, frequently outside their local areas and some have to travel to another country [65, 71, 72]. Significantly, while Poland still lacks rapid a diagnostic and therapeutic path for developmental and epileptic encephalopathy (DEE), 19.8% of parents enrolled in this study reported seeking DS diagnosis abroad. Finally, poor communication with doctors, lack of a whole patient view and fragmented care also contribute to late diagnosis [73].

This survey also shows that, apart from improving access to modern genetic testing, there is an urgent need to raise awareness of rare diseases in routine clinical practice and that academic programmes on rare diseases for doctors should be included in medical education and postgraduate training [66–70]. More emphasis should also be placed on ‘‘thinking genetically” and “genetic red flags” [58, 74], i.e. signs or symptoms that raise the clinical possibility of an underlying genetic disease and taking into account genetic aetiologies in the differential diagnosis of a wide range of diseases that may be encountered in primary care. This is particularly important since gaps in epilepsy awareness and management have been identified and many primary care doctors lack knowledge on developmental and epileptic encephalopathy (DEE) and this may lead to an inaccurate clinical control and referral to a specialist [75]. In fact, a previous study conducted in Poland demonstrated that many general doctors considered themselves to be professionals with limited expertise in rare diseases and believed themselves to be unqualified to treat patients with such conditions [66].

Finally, since approximately 30% of patients with epilepsy fail to respond to drug treatment, in order to qualify difficult epilepsy cases, particularly those that require surgical treatment, a nationwide network of reference centres for epilepsy care needs to be established. It is also suggested that these centres include three levels of care: a neurological clinic, an antiepileptic clinic (a hospital clinic within the neurology department/neurology clinic) and an invasive epilepsy treatment centre. In order to identify as soon as possible and begin tailored treatment from the outset, it is advised that a rapid diagnostic and therapeutic path (the effectiveness of therapy diminishes with the use of another line of treatment) be established and executed.

### Limitations

While to the best of our knowledge, this is, to date, one of the few and one of the largest studies conducted on Polish caregivers of persons with DS, it still includes responses from only 106 caregivers who volunteered and participated in the study. Participants were recruited with the help of the Association for People with Severe Refractory Epilepsy DRAVET.PL via a support group for the DS community on Facebook, so there may be a risk of recruitment bias. These results are also limited by their self-reported, subjective, and retrospective design; consequently, the reported findings are only hypothetical and may be unrepresentative of a well-defined medical assessment. Since this survey was part of a larger study on the experiences of family caregivers of persons with rare diseases with the diagnostic process, it was also impossible to construct one questionnaire to ask many questions that relate specifically to DS. Consequently, because only several general questions about diagnostic tests were asked, a further, in-depth study is recommended. Neither did we examine DS children’s clinical data, including seizures, but focused on caregivers’ experiences with the diagnostic process and their perception of its possible consequences. Meanwhile, since the type, frequency, intensity and severity of seizures affect a child’s health condition, including physical health, intellectual development and communication skills, they may also affect caregivers’ stress levels. Although the questionnaire used in this survey was designed with the help of several experts: a peadiatrician specialising in RDs, a medical sociologist, a public health specialist and the parent of a DS child, it was an ad hoc tool and was not validated. Although opinion data are helpful, as they may help understand caregivers’ perceptions and views, they only represent subjective results. This means that caregivers’ opinions on the diagnostic odyssey and the consequences of delayed or misdiagnoses are based only on what the caregivers reported. Although 17.9% of respondents were fathers, female caregivers still predominated. Finally, more in-depth qualitative research is required to understand caregivers’ experiences with the diagnostic odyssey in DS.

## Conclusions

This study confirms that Polish DS parents experience problems in receiving a timely, accurate diagnosis for their children. It also shows that, as in many other RDs, the diagnostic odyssey and labyrinth in DS result in several misdiagnoses and the need to consult numerous specialists before the final diagnosis is made. It therefore becomes a source of irritability frustration and fear among parents, as it forces them to become their children’s paediatrician, neurologist and genetic consultant [62]. It also shows that, according to DS parents, delayed diagnosis has many damaging health consequences for their children and leads to many unnecessary, invasive and possibly harmful and costly hospitalisations, medical procedures, and medication, which may aggravate the course of the disease.

Since genetic testing usually concludes the diagnostic odyssey [63, 64], this study also highlights the need for widespread access to modern genetic testing. This is of particular importance since early diagnosis is crucial for the management of proper treatment schemes for DS patients, which helps to avoid unnecessary therapies based on sodium channels, control seizures and reduces the risk of prolonged status epilepticus and SUDEP. It is especially important since Poland is currenlty implementing its Rare Diseases Plan for 2024–2025 [65], the aim of which is to improve the situation of RD patients and their families by creating an integrated healthcare model that will enable comprehensive and co-ordinated care by establishing Expert Centres for Rare Diseases (OECR), improving the access to modern genetic diagnostic tests and high-quality, innovative treatment, the creation of the Polish Register of Rare Diseases and Rare Disease Patient Card, and expanding knowledge about rare diseases by running the Rare Diseases Information Platform.

In addition to enhancing access to modern genetic testing, there is also a need to increase general practitioners’ awareness of the DEE [75]. The creation of more rapid and transparent referral procedures for children with DEE in order promptly to identify and initiate personalised treatment from the onset of symptoms needs to be rolled out across the country. Finally, since lack of or inadequate financial and psychological support was another factor identified among caregivers, there is a need for improved communication between patients and doctors, and financial and psychological support, particularly during the diagnosis.

## Data Availability

The data supporting this study’s findings are not openly available due to reasons of sensitivity and are available from the corresponding author upon reasonable request. Data are located in controlled access data storage at Poznan University of Medical Sciences.

## Abbreviations

DEE: Developmental and epileptic encephalopathy
DS: Dravet syndrome
RD: Rare diseases
SUDEP: Sudden unexpected death in epileptic patients
